# High Utilizer Care Plan Project: A Network Initiative to Decrease Inappropriate Resource Utilization Among High-Risk Patients

**DOI:** 10.7759/cureus.100999

**Published:** 2026-01-07

**Authors:** Vanessa Reese, Justin Psaila, Marisa Schwartz, Shawn Owens, Rebecca Miller, Maria Martinez-Baladejo, Wayne Bauerle, Luis Alvarado, Stanislaw Stawicki, Anna Ng-Pellegrino

**Affiliations:** 1 Research and Innovation, St. Luke's University Health Network, Bethlehem, USA; 2 Internal Medicine, St. Luke's University Health Network, Easton, USA; 3 Executive Administration, St. Luke's University Health Network, Bethlehem, USA; 4 Care Management, St. Luke's University Health Network, Allentown, USA; 5 Psychiatry, St. Luke’s University Health Network, Orwigsburg, USA; 6 Surgery, St. Luke's University Health Network, Bethlehem, USA; 7 Graduate Medical Education (GME) Data Measurement and Outcomes Assessment, St. Luke's University Health Network, Bethlehem, USA; 8 Anesthesiology and Perioperative Medicine, St. Luke's University Health Network, Bethlehem, USA

**Keywords:** abusive health care use, healthcare users, high utilizer, individualized care plan, quality improvement study

## Abstract

Introduction: Healthcare costs in the United States continue to rise. Among many factors, this can be attributed to a small subset of the overall patient population, termed “high utilizers” (HUs). HUs account for nearly 50% of health care costs. The costs associated with HUs tend to be disproportionate, often unnecessary, and not associated with improved patient outcomes. The excessive utilization of resources by HUs is multifactorial, requiring innovative and multifaceted solutions. Our institution implemented a comprehensive program to not only identify HUs but also to address the identified underlying clinical and social determinants in order to provide appropriate care while reducing resource overuse. We hypothesized that a robust quality improvement process coupled with individualized care plans (ICPs) would result in effective identification of HUs and decrease associated costs.

Methods: From July 2021 to July 2025, a multidisciplinary team identified patients as HUs based on existing Department of Health and Human Services definitions. The team then created ICPs for each patient. These care plans were linked to the patient’s electronic medical record (EMR) and appeared each time a provider accessed the EMR. The cost per patient and usage of inpatient, outpatient, and Emergency Department (ED) facilities were measured. Aggregate endpoints were captured six months prior to ICP initiation and six months after ICP implementation, and the final endpoint was taken at the last six months of the study, regardless of how long the patient was on their ICP. In addition to descriptive statistics, temporal and group comparisons were performed using STATA software (StataCorp, College Station, TX, USA), with a Bonferroni correction applied and significance set at P<0.017.

Results: A total of 190 patients were enrolled in ICPs over the study period. The mean age was 45.15 years, and the majority of the patients (62.6%) were female sex. Following the implementation of ICPs, there was a $40.91 million reduction in charges over the four-year course of the study. Overall, there were statistically significant decreases in ED visits (14.18 to 6.75 mean visits), inpatient visits (4.43 to 2.41 mean visits), and inpatient imaging (9.35 to 5.41 mean studies) using the beginning of the ICP as a starting point and the most recent six months snapshot as the endpoint.

Conclusion: High utilizers contribute to unacceptably high healthcare expenditures. This study shows that a hybrid approach consisting of a multidisciplinary team, standardized definitions, technology-aided protocols to identify HU, and ICPs effectively reduces costs as well as ED and inpatient visits. This approach also reduces unnecessary imaging.

## Introduction

When the Affordable Care Act (ACA) policy was implemented in the United States (US), payment structure was transitioned from a “fee-for-service” model to an “alternative payment” model geared towards compensation based on patient outcomes [1]. More specifically, the ACA transformed the paradigm so that value-optimization of care delivered, and not just its quality or quantity in isolation, became the goal. These transformational reforms encompass the so-called “triple aim” set of principles, with the corresponding pillars being: improving the experience of the care, improving the health of populations, and reducing the costs associated with medical care [2]. Although there has been a concerted effort by the medical community to contain systemic costs, the US healthcare expenditures continue to rise, currently accounting for nearly 20% of the Gross Domestic Product [3].

Important to our current work, a large portion of the costs recorded by the US healthcare system can be directly attributed to a specific subgroup of patients. The so-called “High Utilizers” (HUs), as described by Siekman et al., are a small, complicated subset of patients whose physical and mental illnesses, interwoven with substance abuse and/or low socioeconomic status, result in substantial medical costs [4]. The Department of Health and Human Services defines HUs as patients with four or more visits for privately insured patients aged one to 64 years, four or more visits for Medicare patients aged 65 years and older, and six or more visits for Medicaid or Medicare patients aged one to 64 years [5]. Constituting about 5% of the overall patient population, HUs account for 40-50% of the total costs [6,7]. Moreover, the top 1% of utilizers alone account for nearly 25% of all healthcare expenditures [4]. It is not unusual for the resources allocated to treat HUs to be redundant, unnecessary, inappropriate, and ineffective in regard to the overall long-term medical management [8]. Unfortunately, these patients often have complex needs, both physical and psychosocial, that tend to be poorly managed by the healthcare system [9]. Addressing the needs of the HU population has the potential for not only reducing healthcare spending, but also concomitantly improving their clinical outcomes and long-term quality of life [10,11]. 

Expanding upon this team’s ongoing work on utilizing individualized care plans (ICPs), the purpose of the current study was to incorporate a multidisciplinary approach when creating ICPs while simultaneously making the ICPs easily accessible to various medical providers through the hospital’s electronic medical record (EMR). ICPs are treatment plans that incorporate the medical diagnoses and needs specific to the patient as well as their social determinants of health [12]. The study's primary objective was to reduce emergency department (ED) encounters, inpatient admissions, inpatient and outpatient radiological studies, and care-related costs within our health system, while redirecting care to an appropriate setting. We hypothesized that a robust quality improvement process coupled with ICPs would result in better identification of HUs and a decrease in inappropriate overuse of resources within this population.

The abstract of this article was previously presented as a poster at the 2025 Keystone Chapter ACS Spring conference on May 2, 2025.

## Materials and methods

The setting of this study is a large, 16-hospital regional health network in Eastern Pennsylvania, USA. Institutional Review Board (IRB) exemption was granted (SLQI2022-75) before proceeding with study activities. The manuscript was prepared according to SQUIRE guidelines [13]. In 2019, within the health network, a multi-disciplinary High Utilizer Care Plan Team (HUCPT) was created, consisting of 15 professionals, representing the following areas: ED physicians, hospitalists, care managers, parish nurses (a nurse who incorporates faith-based practices into their nursing practice), administrators, and a clinical informatics nurse. A patient-specific, hybrid treatment approach was developed using standardized HU definitions, resources, and modalities, but also including ICPs unique to each patient’s needs. All patients received appropriate medical treatment (consistent with the standard of care) and were provided with a care team consisting of bedside providers and case management, along with required community resources to alleviate health-related social needs, as indicated. The customizable portions of the plan revolved around the social and behavioral barriers specific to each patient.

Identification of subjects

The study team reviewed the network EMR to identify patients with the highest number of ED visits from July 2021 through July 2024. Data from 2019 - 2020 was not collected due to low numbers for the initial implementation from the COVID-19 pandemic, which would skew the data. This review included all internal ED visits (within our hospital network) and external visits (outside of our hospital network) that were visible within our health information exchange (HIE). Initially, this process was largely referral-based. Patients suspected of being HUs were referred to the committee by physicians and health care providers for review. A designated advanced practice clinician specializing in Hospital Medicine subsequently screened the EMR and identified the patient in question as an HU when there were more than three to six visits to the ED for the same complaints within a six-month period, resulting in subsequent admissions and duplicative/redundant diagnostic testing. These criteria closely approximated the Department of Health and Human Services' definitions of HUs. Exclusion criteria included medically necessary treatment and follow-up visits within the six to 12 months of high utilization, patients with substance abuse disorders who refused rehabilitation, and patients receiving hospice care. 

Individualized care plan creation

**Figure 1:**
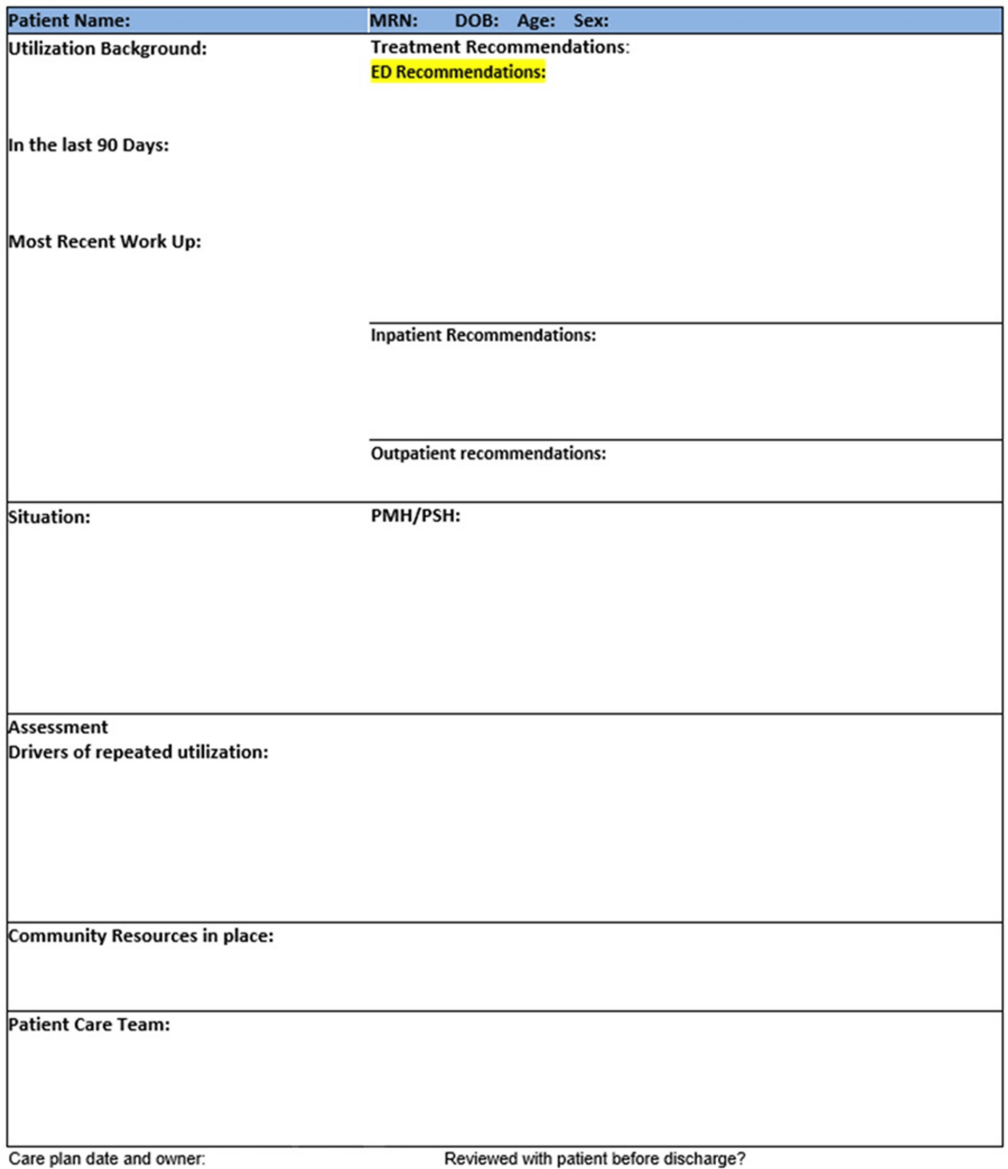
Sample Individualized Care Plan PMH: past medical history, PSH: past surgical history

Once a patient was determined to be a HU, a designated clinician reviewed the patient’s medical and surgical history, prior workup, baseline lab studies, imaging frequency and results, home medication lists, and notations from outpatient providers. This medical provider then formulated an ICP for each of the identified patients based on the clinical and social determinants of health (Figure 1). Components of the ICP include the patient’s 90-day utilization, recent work-up and imaging, past medical/surgical history, treatment recommendations in the ED, outpatient, and inpatient setting, drivers of repeat utilization, identification of community resources in place, and the patient care team in place. This process also incorporated input and review by the patient’s primary care provider (PCP) as well as any pertinent specialist(s) concerning any further management recommendations.

For cases solely involving patients using the ED for routine visits more applicable to the ambulatory primary care or urgent care settings, the care manager would arrange outpatient follow-up and connect the patient to network or community services, with necessary coordination provided. These patients would be placed on a curated watch-list rather than becoming immediately flagged as HU; under such circumstances, no immediate care plan would be created.

Following the development of an ICP, a review by the HUCPT is conducted. After approval of the proposed plan, the specific patient in question was contacted by the medical provider who created the plan. Subsequent communications included the disclosure of the existence of an ICP, its visibility to all the providers in the network, as well as the provision of detailed explanation and justification for said plan. Patients were also reassured that the intervention is not punitive in nature, but rather a measure to ensure they receive appropriate, safe, and timely care. Individual plans and the watch-list were re-evaluated every 90 days and either continued, updated, or labeled as surveillance, as determined during the standardized review process. The standardized review process involved evaluation for mortalities, admissions/readmissions, ED visits, length of stay for each admission, social determinants of health, and change (increase or decrease) in utilization.

Criteria for moving a patient to surveillance rather than active care management on an ICP (i.e. update or continuation) include: (A) no utilization in the last three to six months; (B) compliance with primary care follow up; (C) the attainment of goals on social determinants of health; (D) patient ability to self-manage their disease process. Patients who were marked as surveillance still have their ICP active in the EPIC system, but the follow-up from case management is less active and frequent.

Flagging of high utilizer patients

Upon the finalization of each patient agreement, the detailed plan was then uploaded to the EMR and a flag titled “High Utilizer” was added, along with a best practice advisory (BPA) containing a hyperlink to the ICP. Both the flag and the BPA appear automatically upon opening the patient’s EMR, along with acknowledgement messaging. Three options are provided along with the message; “I have read the action plan,” “I’ll get to it,” or “I am not the attending provider.” If “I’ll get to it” was selected, the flag and acknowledgement message would automatically reappear in one hour. If the provider indicated “I have read the action plan,” or that “I am not the attending physician,” the BPA will not be displayed again for that user during the ensuing 24 hours. A free text field was also provided for the clinician to write-in customized comments.

The above tools were not initially incorporated into the Network’s EMR framework and had to be custom-built by the medical informatics team. Once built, the new framework was piloted by the HUCPT team utilizing a cohort of pre-selected patients prior to network-wide launch. Once the tool was ready for launch, education was provided to stakeholder clinical teams, including our EDs, hospitalists, and PCPs. Clinicians who subsequently joined the network were given the ICP education to ensure that there was continued adherence to the care plan, as well as ongoing understanding of the team’s goals across all participating providers. Provider compliance with reviewing the care plan when the BPA fires was monitored, along with measuring care plan utilization in six-month intervals. The first measurements were taken six months prior to implementation of ICP, six months post ICP implementation, and the last six months of the study. All timepoints were collected on a rolling basis and aggregated for analysis.

Endpoints

We reviewed all patients who spent at least six months within the HU cycle between July 2021 and July 2024 and determined the effects of current intervention on health network per-patient costs and numerous endpoints. As enrollment of HU patients occurred continuously, endpoints were measured on a rolling basis as each patient arrived at the predetermined timepoint. The endpoints were then aggregated together. Individual endpoints were captured six months prior to care plan initiation (prior), six months after care plan implementation (next), and the last six months of the study (recent), regardless of how long the patient had been enrolled in their ICP. Primary endpoints measured included ED encounters, inpatient admissions, inpatient radiologic studies, and the associated cost(s) of care. Costs were based on the total cost incurred by the network for ED visits, hospital admissions or observations, imaging studies, and outpatient hospital encounters related to HUs combined. Imaging included ultrasound, CT, X-ray, nuclear medicine, mammography, Dexa scans, and MRI charges. Outpatient hospital encounters were urgent care, outpatient testing, physical/occupational/ speech therapy, and hospital-based clinic encounters. Number of outpatient visits and outpatient radiologic studies were measured as secondary endpoints. Demographic data was also collected from the EMR for comparison to the national population.

Statistical analysis

The data were summarized using the median, interquartile range (IQR), mean, and standard deviation (SD) for continuous variables, and frequency and percentage for categorical variables. Comparisons were made across three time points: prior, next, and recent. A Bonferroni correction was applied to minimize the risk of false positives across multiple statistical tests (P < 0.017). Wilcoxon matched-pairs signed-rank test and paired T-test were conducted to assess the median and average differences respectively in hospital services between the time points. The median difference and the 95% confidence intervals for percentile differences were calculated and presented. P-values were considered statistically significant if they were less than 0.017. All data analysis was performed using STATA V18 (StataCorp LLC, College Station, TX, USA).

## Results

A total of 190 patients were enrolled in the HU program during the study period. The mean age of the study population was 45.15 years. The population was predominantly female sex (37.37% male and 62.63% female). The racial and ethnic composition of the population was 71.58% White, 15.26% Black, 13.16% other races such as Native American, Asian, etc., and 17.37% were of Hispanic ethnicity. When evaluating payor index, the majority of the patients were on government-funded insurance, such as Medicare/Medicare Advantage (40%) and Medicaid/managed Medicaid (39.47%), versus 10% with some form of commercial insurance (Table 1).

**Table 1:** Summary of demographic and insurance variables of interest Data presented as descriptive statistics using mean and standard deviations of the total population. N: total population, SD: standard deviation

Factor	Overall N (%)
N	190
AGE, mean (SD)	45.15 (13.88)
SEX	
Male	71 (37.37%)
Female	119 (62.63%)
RACE	
American Indian or Alaska Native	1 (0.53%)
Black or African American	29 (15.26%)
Mixed Race	5 (2.63%)
Native Hawaiian/Pacific Islander	1 (0.53%)
Other Race	18 (9.47%)
White or Caucasian	136 (71.58%)
HISPANIC	
No	157 (82.63%)
Yes	33 (17.37%)
INSURER	
Medicare	76 (40.00%)
Medicaid	75 (39.47%)
Commercial/Private	19 (10.00%)
Unknown	14 (7.37%)
Self-pay/Uninsured	4 (2.11%)
Federal	1 (0.53%)
Charity	1 (0.53%)

Primary outcomes

Our primary outcomes were in-network ED encounters, inpatient admissions, and inpatient radiologic studies. At the next timepoint, there was a significant reduction in ED visits (9.46 mean visits compared to 13.16 prior, p <0.0001), inpatient admissions (2.62 mean visits versus 4.30 prior, p <0.0001), and inpatient radiological studies (5.62 mean studies compared to 8.83 prior, p <0.0001). This is demonstrated in Table 2. The reduction in resource utilization was greater the longer the patients were participants in their ICP. During the recent timepoint, the mean ED visits were 6.75 (p <0.0001), mean inpatient visits were 2.41 (p <0.0001), and mean inpatient radiological studies were 5.41 (p = 0.0006) (Table 3).

**Table 2:** Pairwise comparison of hospital services used across care plan initiation time points (prior to ICP vs next six months with ICP) Data are presented as N of the total cohort, N = 190 with occurrences for both timepoints being compared. Statistical comparisons were performed using a paired T-test and a Wilcoxon matched-pairs signed-rank test with a Bonferroni correction value. P-values were considered significant at a value less than 0.017 (Bonferroni correction). * The log-transformed values were analyzed. ^a ^T-statistic ^b ^Percentile difference with 95% confidence interval ED: emergency department; ICP: individualized care plan; IP: inpatient; OP: outpatient; Img: imaging; PCP: primary care physician; SD: standard deviation; IQR: interquartile range

Factor	N	Prior	Next	Statistic	p-value
ED Visits					
Mean (SD)*	145	13.16 (13.5)	9.46 (13.26)	7.56^a^	<0.0001
Median (IQR)	145	11 (7, 15)	7 (3, 11)	4 (2, 5)^b^	<0.001
IP Visits					
Mean (SD)*	66	4.30 (2.49)	2.62 (1.98)	6.83^a^	<0.0001
Median (IQR)		4 (3, 5)	2 (1, 4)	2 (1, 2)^b^	<0.001
OP Visits	---	---	---	---	---
IP Image Orders					
Mean (SD)*	61	8.83 (6.41)	5.62 (5.26)	4.41^a^	<0.0001
Median (IQR)	61	7 (4, 12)	4 (2, 7)	3 (1, 5)^b^	<0.001
OP Image Orders					
Mean (SD)*	125	10.90 (12.96)	12.51 (16.21)	0.96^a^	0.336
Median (IQR)	125	6 (4, 13)	6 (3, 16)	1 (-1, 2)^b^	0.841
Hospital Charges	---	---	---	---	---
PCP Visits					
Mean (SD)*	63	4.38 (3.03)	6.65 (2.57)	1.42^a^	0.162
Median (IQR)	63	3 (2, 6)	3 (2, 5)	0 (0, 1)^b^	0.021
Specialist Visits					
Mean (SD)*	103	5.31 (5.33)	4.99 (6.14)	0.78^a^	0.433
Median (IQR)	103	4 (2, 7)	3 (2, 6)	0 (0, 1)^b^	0.215

**Table 3:** Pairwise comparison of hospital services used across care plan initiation time points (prior to ICP vs recent six months after ICP implementation) Data are presented as N of the total cohort, N = 190 with occurrences for both timepoints being compared. Statistical comparisons were performed using a paired T-test and a Wilcoxon matched-pairs signed-rank test with a Bonferroni correction value. P-values were considered significant at a value less than 0.017 (Bonferroni correction). * The log-transformed values were analyzed. ^a^ T-statistic ^b^ Percentile difference with 95% confidence interval ICP: individualized care plan; ED: emergency department; IP: inpatient; OP: outpatient; Img: imaging; PCP: primary care physician; SD: standard deviation, IQR: interquartile range

Factor	N	Prior	Recent	Statistic	p-value*
ED Visits					
Mean (SD)*	116	14.18 (14.79)	6.75 (7.59)	9.05^a^	<0.0001
Median (IQR)	116	11.5 (6.5, 17)	4 (2, 8)	6 (4, 8)^b^	<0.001
IP Visits					
Mean (SD)*	51	4.43 (3.06)	2.41 (1.96)	6.01^a^	<0.0001
Median (IQR)	51	4 (2, 5)	2 (1,3)	2 (1, 2)^b^	<0.001
OP Visits	---	---	---	---	---
IP Image Orders					
Mean (SD)*	49	9.35 (8.73)	5.41 (5.89)	3.68^a^	0.0006
Median (IQR)	49	8 (4, 12)	4 (2, 6)	3 (1, 5)^b^	<0.001
OP Image Orders					
Mean (SD)*	114	10.57 (12.97)	9.44 (13.22)	2.78^a^	0.006
Median (IQR)	114	6 (4, 112)	5 (2, 11)	2 (0, 3)^b^	0.079
Hospital Charges	---	---	---	---	---
PCP Visits					
Mean (SD)*	52	3.83 (2.67)	2.81 (2.07)	2.22^a^	0.031
Median (IQR)	52	3 (2, 5)	2 (2, 3)	1 (0, 2)^b^	0.003
Specialist Visits					
Mean (SD)*	83	5.94 (5.56)	5.24 (4.89)	1.23^a^	0.224
Median (IQR)	83	4 (2, 8)	3 (2, 7)	0 (0, 1)^b^	0.105

Secondary outcomes

Outpatient visits and outpatient radiographic studies were the secondary outcomes of interest. Regarding the secondary outcomes, at the next timepoint, outpatient visits increased by 39.02% (from 469 to 652 visits) without a statistically significant increase in outpatient radiological studies ordered. For the recent timepoint, there were 627 outpatient visits, representing a 33.69% increase compared to prior levels. There was also a significant reduction in outpatient radiological studies, dropping from 10.57 mean imaging orders to 9.44 (p = 0.006) (Table 3). 

Hospital cost

**Figure 2:**
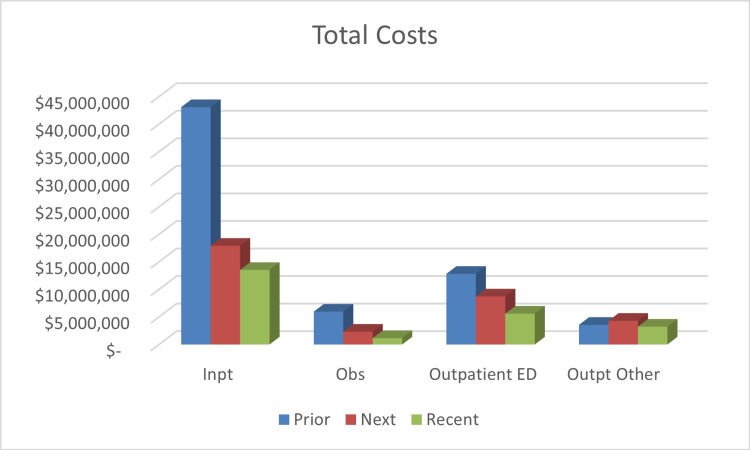
Total Hospital Costs Cost is represented in US dollars. Prior: six months before the start of the individualized care plan; Next: six months after the start of the individualized care plan; Recent: last six months of the study Inpt: inpatient; Obs: observation; ED: emergency department; Outpt: outpatient; mo: months; ICP: Individualized care plan

**Figure 3:**
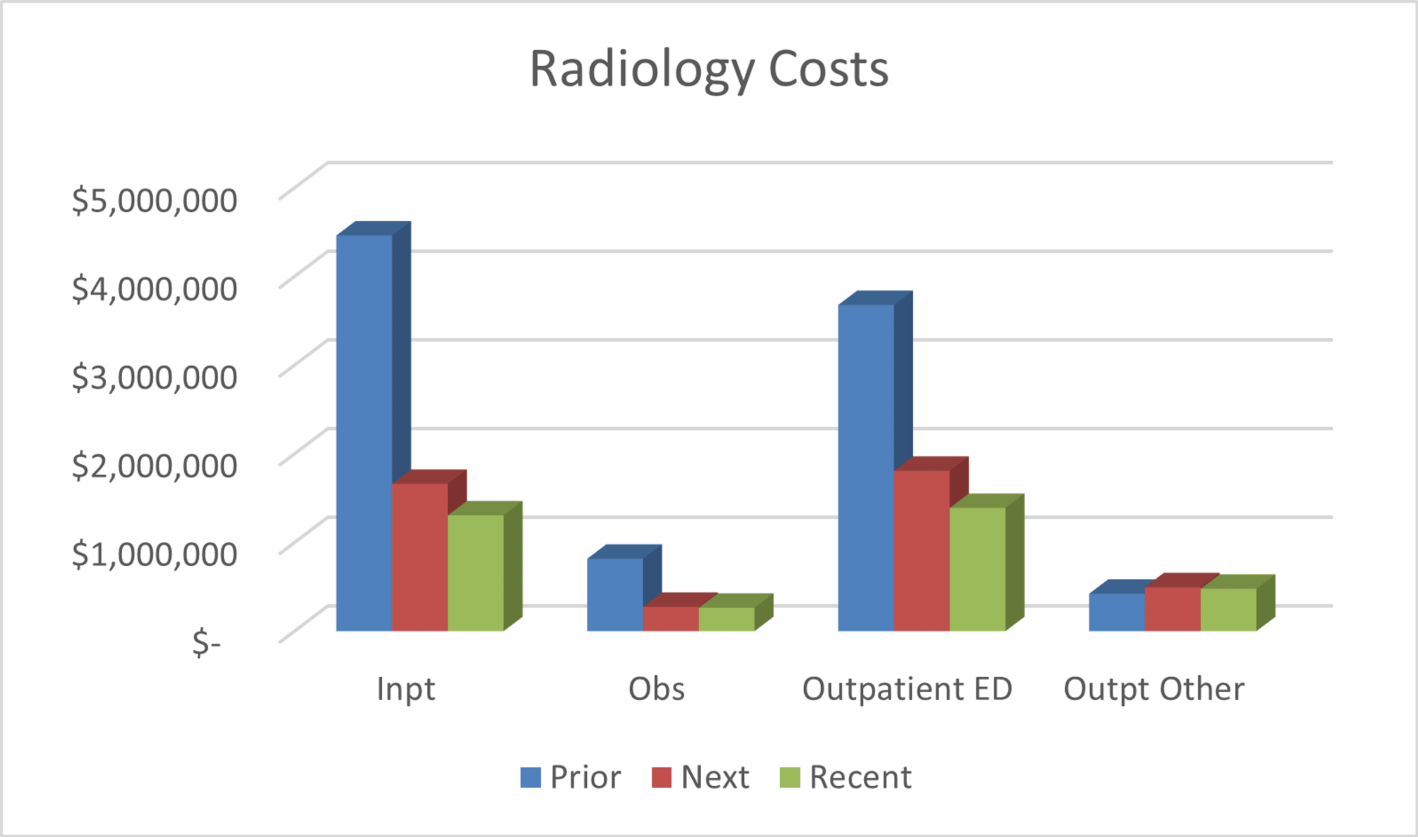
Summary of Radiology Charges Cost is represented in US dollars. Prior: six months before the start of the individualized care plan; Next: six months after the start of the individualized care plan; Recent: last six months of the study Inpt: inpatient; Obs: observation; ED: emergency department; Outpt: outpatient; mo: months; ICP: Individualized care plan

**Figure 4:**
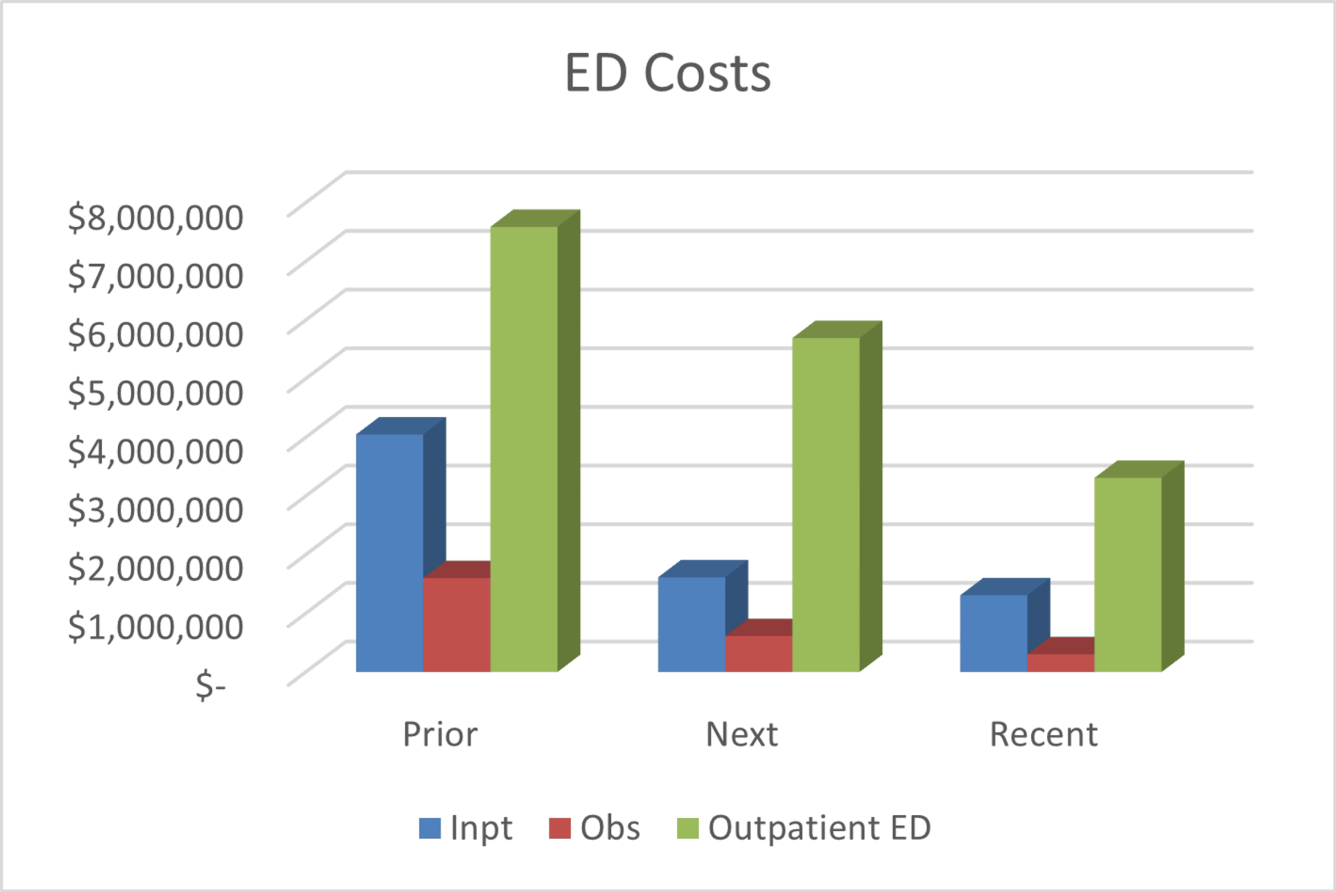
Summary of Emergency Department Cost (In Hospital Network) Cost is represented in US dollars. Prior: six months before the start of the individualized care plan; Next: six months after the start of the individualized care plan; Recent: last six months of the study Inpt: inpatient; Obs: observation; ED: emergency department; Outpt: outpatient; mo: months; ICP: Individualized care plan

Total cost for care had been reduced by 48.4% (from $65.44 million to $33.28 million) following six months of care plan utilization, saving $31.16 million. In the recent timepoint, costs were reduced by 65.3% (from $64.44 million to $23.54 million), resulting in approximately $40.91 million in savings. Figures 2-4 offer a breakdown of the cost per endpoint. Specifically, the inpatient admission charges decreased from $43.08 million prior to $17.92 million at the next timepoint (a 58.4% reduction). Inpatient charges were $13.53 million for the recent timepoint (68.6% decrease). Costs associated with inpatient radiologic studies dropped by 58.2% after six months of ICP use ($8.96 million prior, $3.74 million next). At the recent timepoint, costs from inpatient imaging were $2.96 million, a 67% decrease. ED charges were $13.24 million prior and decreased to $7.92 million next, which is a 40.2% reduction. During the recent timepoint, the charges had decreased to $4.92 million, equating to a 62.8% reduction.

## Discussion

A comprehensive multi-tiered approach to identify and individualize the care of HUs resulted in lower resource utilization and costs while providing safe, quality care. This has increasing importance in the era of value-based care and diminished reimbursements, coupled with rising healthcare costs. Considering HUs potentially account for up to $2.15 billion (50% of the $4.3 billion spent on health care in 2021) [14], reducing expenditures for this population holds the potential to markedly decrease spending nationwide. The initial increase in outpatient utilization during the first six months of program implementation can be attributed to patients undergoing initial evaluations and follow-ups that naturally required more than one visit to the physician. Subsequently, once the patient's complex conditions were stabilized, there were fewer required follow-up appointments. This, in turn, resulted in lower utilization of outpatient services, without concomitant increase in ED encounters at the 12 month and beyond intervals.

Several methods to improve the delivery of health services for HUs have been described, ranging from the use of administration data in EMRs to identify HUs to case management and personalized visits in the outpatient setting [15-17]. For the proper medical management of HUs, a multidisciplinary approach is essential to determine the resources required to provide adequate care and to address the patient’s needs. In order to accomplish this task, individualized multidisciplinary care plans for HUs have been proposed as a strategy [18]. More specifically, the care plan must outline specific treatment recommendations, and it must be presented in an algorithmic manner. To optimize the benefits of such a care plan, a dedicated committee should evaluate its effectiveness, assess patient and provider adherence, and review the care plan frequently [19]. 

Multiple studies used ICPs or case management in isolation to reduce high utilization with results that support our findings. Mercer et al. implemented ICPs for HUs and achieved a 50.5% reduction in hospital admissions, a 51.5% decrease in 30-day re-admissions, and a 35.8% reduction in direct costs, all of which were completed in a 12-month timespan [20]. A randomized controlled trial by Seaberg et al. found that “patient navigator” facilitated assessments of complex healthcare needs of HUs reduced overall costs (approximately $800,000 reduction over the one-year post intervention period), decreased hospital admissions and ED visits, and increased PCP use [21]. Likewise, Okin et al. found a reduction in ED visits and a median $132,726 cost reduction in a 12-month study of dedicated case management (CM) for HUs [22]. A report by Linkins et al. of six California pilot programs with a similar study design found that patients receiving CM assistance had a statistically significant reduction in ED utilization and hospital cost during the first year of enlistment. The aggregated cost reduction across the six pilot programs was $1.47 million in ED costs and $2.72 million in inpatient expenses [23]. Similar to our study, the Linkins group found that the longer the patients were enrolled in the program, the greater the decrease was in ED visits [23].

A combined approach of ICPs and social work/case management in an outpatient setting was performed by Dr. Jeffrey Brenner [24]. This resulted in a 40% reduction in emergency department visits and inpatient admissions, and a 56% reduction in medical costs [25]. This was accomplished within a small cohort of 36 patients. Though the setting of the Brenner study differs from the multicenter health network of this study, this supports our hypothesis that a multimodal approach to the identification and care of HUs results in decreased utilization.

The uniqueness of our study is a combined utilization of case management, social work, and ICPs, all within a large network of community hospitals. The $40.91 million reduction in attributable charges throughout our hospital network over the four-year course of the study represents one of the larger decreases in expenditures when compared to savings reported in the literature for similar programs. Given this, our program could reasonably serve as a model for other healthcare systems.

In terms of broader applicability of our findings, the demographics for this patient population were generalizable to the larger US population. The nationwide demographics for 2022 shows 75.8% of the population is White, 13.6% is Black, and 18.9% of the population is of Hispanic ethnicity [25]. The higher proportion of HUs with Medicare or Medicaid insurance coverage is similar to other studies, but contrasts with the nationwide payor index of 66% of the population maintaining private insurance [21,22,26].

Limitations 

The limitations of this study encompass the planning and design aspects, compliance, and uncontrollable system/patient factors. This is a retrospective review of a prospectively conducted quality improvement project with a small sample size and no control group. Given the nature of this study we were unable to adjust for potential confounding factors such as age, comorbidities, or socioeconomic variables. Also, patients were initially referred to the program introducing the potential for underreporting of HUs (it may be difficult to identify patients throughout multiple locations) as well as selection, recall, or misclassification biases. One approach to resolve this would be the implementation of an automated algorithm to automatically identify and refer potential HU patients for review. The cost analysis did not factor in the costs of outpatient visits. Being that these visits increased over the study period, it can be surmised that an increase in the outpatient cost may offset some of the total cost savings seen.

Compliance with the care plan and utility of the care plan for subsequent visits of each HU were not measured, as there is no tool to accomplish this built into the workflow. This makes it hard to completely establish the impact of the plan itself and ensure adherence in clinically relevant situations.

There were several patient-specific considerations that led to limitations in the study. Firstly, some patients were referred to the program in the end stages of their disease. Although most of these patients' use of healthcare was deemed appropriate, they were included in the HU management committee in order to connect these patients with the best available resources and flagged as a rising HU. Other patients required interventions that were not available within the network. Thus, professional relationships were developed with other local care management teams to refer these patients while monitoring their healthcare utilization within the network. Additionally, some patients were lost to follow-up or moved outside of the state of Pennsylvania. It is also difficult to account for the healthcare visits performed at a hospital or offices outside the network. This might introduce inaccuracies in the study as there are several urgent care facilities and EDs in the surrounding area. With the addition of Care Everywhere (Epic Systems, Verona, WI, USA) (software that integrates limited information regarding care received at other facilities), visibility into visits outside the network has increased. To address and track potential high utilization at other facilities, the HUCPT reviews the EMR for outside visits.

## Conclusions

Increased health care utilization by a very small proportion of the overall population continues to be a significant issue and strain on resources for both health care entities and, more importantly, the patients themselves. To date, no study has identified a root cause or solid solution, and the cause of high utilization is variable and multitiered. We recommend a hybrid approach with a standardized framework to address the most common contributors to high utilization that also includes customizable elements specific to the patients’ needs. More robust research is needed in this realm, specifically, well-designed multicenter randomized trials in order to confidently draw further conclusions.
